# Alzheimer's disease and gut-brain axis: *Drosophila melanogaster* as a model

**DOI:** 10.3389/fnins.2025.1543826

**Published:** 2025-02-04

**Authors:** Samuel de Mattos Alves, Paulo Noronha Lisboa-Filho, Carolina Letícia Zilli Vieira, Marina Piacenti-Silva

**Affiliations:** ^1^Institute of Biosciences of Botucatu, Campus Botucatu, São Paulo State University (UNESP), Botucatu, SP, Brazil; ^2^School of Sciences, Campus Bauru, São Paulo State University (UNESP), Bauru, SP, Brazil; ^3^Harvard T. Chan School of Public Health, Harvard University, Boston, MA, United States

**Keywords:** Alzheimer's disease, *Drosophila melanogaster*, gut-brain axis, microbiota, neurodegeneration

## Abstract

Research indicates that by 2050, more than 150 million people will be living with Alzheimer's disease (AD), a condition associated with neurodegeneration due to the accumulation of amyloid-beta and tau proteins. In addition to genetic background, endocrine disruption, and cellular senescence, management of the gut microbiota has emerged as a key element in the diagnosis, progression, and treatment of AD, as certain bacterial metabolites can travel through the bloodstream and cross the blood-brain barrier. This mini-review explores the relationship between tau protein accumulation and gut dysbiosis in *Drosophila melanogaster*. This model facilitates the investigation of how gut-derived metabolites contribute to neurocognitive impairment and dementia. Understanding the role of direct and indirect bacterial by-products, such as lactate and acetate, in glial cell activation and tau protein dynamics may provide insights into the mechanisms of AD progression and contribute to more effective treatments. Here we discuss how the simplicity and extensive genetic tools of *Drosophila* make it a valuable model for studying these interactions and testing potential therapeutics, including probiotics. Integrating *Drosophila* studies with other established models may reveal conserved pathways and accelerate the translation of findings into clinical applications.

## 1 Introduction

The incidence of Alzheimer's disease (AD) has notably increased in recent years. The number of patients is projected to triple by 2050 (Scheltens et al., 2021), causing not only suffering to family, friends and caregivers (Beata et al., 2023), but deeply consequences to health systems. The condition is strongly associated to the accumulation of amyloid-beta (Aβ) and tau proteins (Palmqvist et al., 2021; Hou et al., 2019; Rydbom et al., 2021), but a secondary approach leveraging the influence of the intestinal tract on the brain has been established (Vogt et al., 2017; Pluta et al., 2020). The modulation of the microorganisms found in the gut showed a new path to treat the disease, since the molecules produced by the microbiota can reach neurons and glial cells and influence them in several ways (Huang et al., 2023; Mayneris-Perxachs et al., 2022).

In addition to the traditional murine models, *Drosophila melanogaster* is an inexpensive, genetically modulable and easily reproducible model (Jennings, 2011) that is currently used in AD and microbiota-related research (Rydbom et al., 2021; Kong et al., 2018; Tan et al., 2020). In addition, the molecular and cellular conserved aspects of *Drosophila* support its use in intestinal epithelium (Apidianakis and Rahme, 2011), and brain-gut communication research (Makdissi et al., 2023; Kitani-Morii et al., 2021), encouraging new AD diagnosis insights from an interorgan perspective.

Aspects of *Drosophila* gut microbiota are common with humans, including bacterial genera such as *Acetobacter* and *Lactobacillus* (Simhadri et al., 2017), which produce acetic acid and lactic acid, respectively. The undissociated form of acetic acid, acetate, presents anti-neuroinflammatory properties (Liu et al., 2020), while lactate modulates aging in flies (Long et al., 2020) and is transported from glial cells to neurons, where it is utilized in the tricarboxylic acid cycle (Xu et al., 2023; Volkenhoff et al., 2015). These organisms play a fundamental role in disrupted energy metabolism associated with AD.

**Figure 1:**
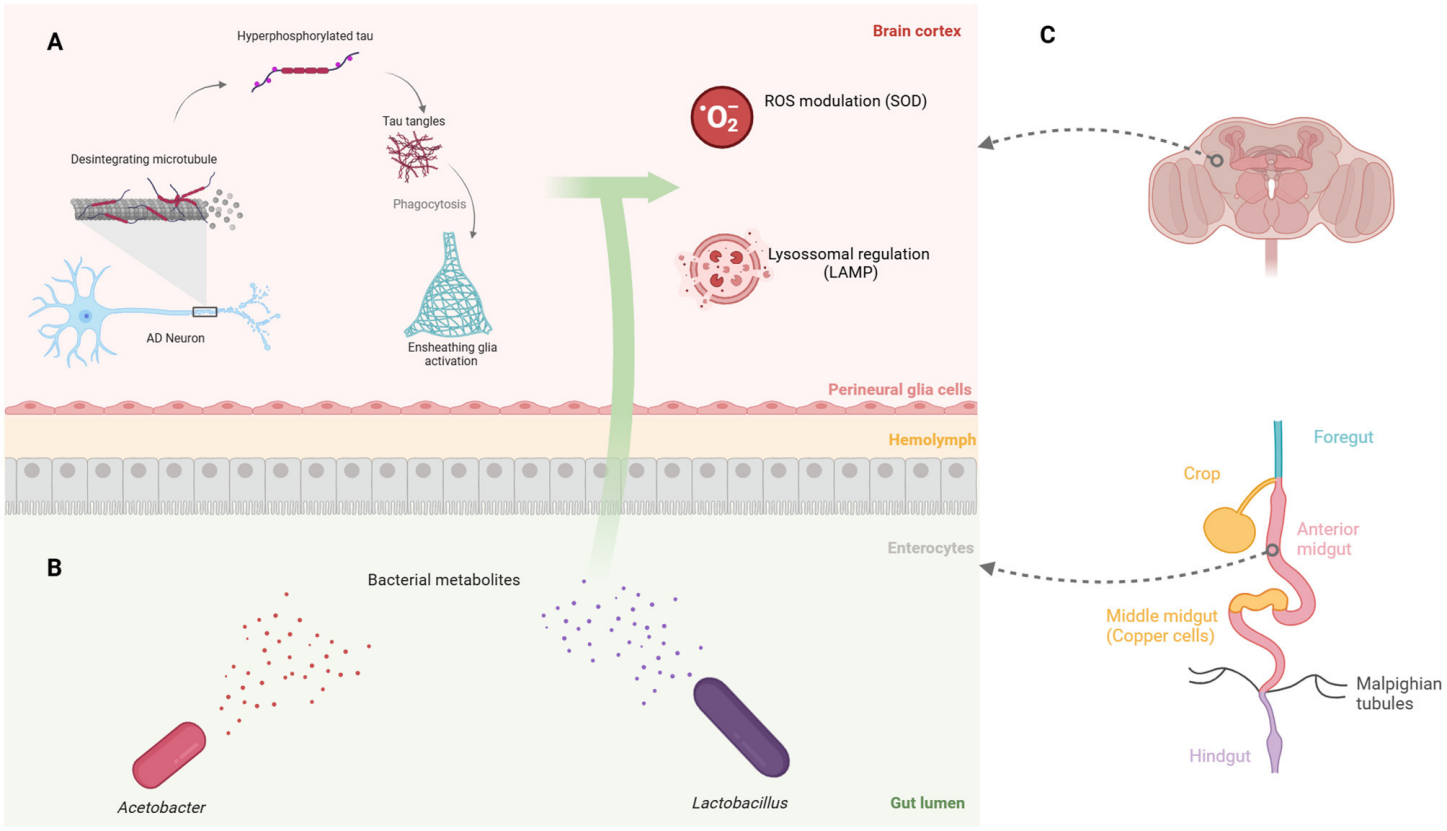
Gut bacterial metabolites in *Drosophila* affect brain processes associated with Alzheimer's disease. **(A)** Engulfment of tau fragments by *Drosophila* ensheathing glia is linked to ROS production and lysosomal activity, both of which may be modulated by gut bacterial metabolites. **(B)** The two primary gut bacteria genera shared between flies and humans: gram-negative *Acetobacter* and gram-positive *Lactobacillus*. **(C)** The gastrointestinal regions of *Drosophila*, highlighting the low pH region of copper cells in the middle midgut, which influences the microbiota composition. Created in https://BioRender.com.

Glial cell types such as microglia participates in tau protein engulfment and neuroprotection in both zebrafish (Hassan-Abdi et al., 2019) and mammals (Freeman and Doherty, 2006; Yildirim et al., 2019). Since its *Drosophila* flies' counterparts—neuropil, cortex, and ensheathing glia (Freeman and Doherty, 2006; Doherty et al., 2009)—also play a role in tau phagocytosis, the activation of ensheathing glia is believed to be crucial for elucidating the pathways involved in tau protein generation (Figure 1A).

Exploring the impact of gut-derived lactate and acetate on AD progression and glial activation in fly models can provide valuable insights into how bacterial by-products modulate neuro-cognitive and homeostatic functions, ultimately guiding more effective treatments for the disease. This mini-review highlights the potential of *Drosophila* as a robust model for investigating the associations between the flies' gut microbiota and the human microbiota, a connection that helps uncover the mechanisms linking bacterial balance to AD progression and inform future therapeutics.

## 2 Proteins associated with Alzheimer's disease

Aβ and tau proteins are the molecules more frequently associated to Alzheimer's disease progression (Lei et al., 2021; Scheltens et al., 2021; Panza et al., 2019). Tau protein is found in neuronal cells of the central nervous system (CNS), mainly in dendrites and axons regions (Rawat et al., 2022), and a diversity of post-translational modifications can cause its abnormal function (Giong et al., 2021). The excessive phosphorylation of tau protein by enzymes known as kinases destabilizes it, making it prone to detaching from microtubules, organelles essential for transporting vesicles and molecules throughout neurons.

Specific regions of tau can be abnormally phosphorylated or, more precisely, hyperphosphorylated. Serine, tyrosine and threonine are the amino acids where this addition occurs, and depending on the position of the phosphate attachment, numerous variants are formed. For instance, tau hyperphosphorylated on threonine 181 is found in blood, which optimize its use as an easy-to-collect biomarker in AD diagnosis (Thijssen et al., 2020), whereas the deficiency of super oxide dismutase 2 (SOD2) exacerbate the levels of tau hyperphosphorylated on serine 396 in mice (Flynn and Melov, 2013; Melov et al., 2007).

Tau phosphorylation introduces a negatively charged phosphate group to the peptide, changing its electrostatics and making it more hydrophilic (Alquezar et al., 2021). The pathological phosphorylation along with the diminished clearance of tau fragments by glial cells and neuroinflammation trigger the formation of insoluble paired helical filaments (Rawat et al., 2022).

The expression of the *Lamp1* gene (lysosomal-associated membrane protein) is decreased in *Drosophila* fruit flies expressing proteins related to Parkinson's, indicating that lysosomal degradative activity plays a crucial role in protecting against oxidative stress and locomotor deficits (Rahmani et al., 2022). Additionally, *Lamp1* is down-regulated in flies expressing Aβ while being up-regulated in models of amyloid- β precursor protein (AβPP) (Bergkvist et al., 2020). This suggests that these vesicles regulate the degradation and toxicity of Aβ oligomers, with significant implications for tau pathology. In contrast, *Lamp2* mutant mice are more severely affected by vacuole formation compared to *Lamp1* (Chaudhry et al., 2022; Rahmani et al., 2022), indicating that their respective alleles operate through different mechanisms across species. Nevertheless, both isoforms are recognized as equally significant biomarkers in the context of neurodegenerative research.

## 3 Gut microbiota and Alzheimer's disease

The interconnection between diet, microbiota, and the intestinal epithelium offers valuable insights into brain health. The gastrointestinal tract engages in a complex bidirectional communication with the nervous system through a sophisticated network of signaling pathways (Makdissi et al., 2023). In mammals, the gut microbiota influences the development of the newborn immune system (Donald and Finlay, 2023), the differentiation of anti-inflammatory T_reg_ cells (Arpaia et al., 2013), hormone levels, neurotransmitter metabolism, neuronal signaling (Morais et al., 2021), and the integrity of blood-brain barrier (Fung et al., 2017). However, the mechanisms through which the intestinal host-microbiota interactions remotely alter brain physiology remain an area of ongoing research (Fung et al., 2017), especially in invertebrate models.

A wide range of bacterial genera perform gut-related functions. *Lactobacillus rhamnosus* modulates the levels of the inhibitory neurotransmitter γ-aminobutyric acid, also known as GABA (Barrett et al., 2012), leading to the regulation of anxiety and depression both in mice (Bravo et al., 2011; Tsai et al., 2023) and humans (Slykerman et al., 2017). Moreover, psychological stress increases the abundance of the gut commensal *L. murinus* in mice, a producer of indole-3-acetate (IAA), which contributes to the loss of intestinal secretory cells (Wei et al., 2024). In addition, *Lactobacillus* shows an intrinsic positive metabolic interplay with *Acetobacter* strains (Dodge et al., 2023), that are equally reduced in neurodegenerative diseases (Liu et al., 2023). The interaction between these groups encourages further studies on how bacterial metabolites may influence neurological diseases (Figure 1B), especially given the diversity of these molecules, which tends to decline with age and the progression of AD (Kong et al., 2018; Lynn et al., 2022).

Some studies have shown that the microbiota can be modified or improved to protect patients against the neurocognitive decline. Instead of administering isolated species such as *Lactobacillus* (Kleerebezem et al., 2010), the solution may lie in fostering an optimal gut—and external—environment that promotes the growth of beneficial bacteria, while also considering their key metabolites. Even social interactions seem to play a role in microbiome-associated diseases (Valles-Colomer et al., 2023). In this context, transplantation of feces from human with AD to germ-free mice decreases the abundance of nervous system mediators, including GABA, taurine, and valine (Fujii et al., 2019). Additionally, the fecal microbiome of patients with the disease exhibits increased levels of *Bacteroidetes*, and decreased levels of *Firmicutes* and *Bifidobacterium* (Vogt et al., 2017), reinforcing the synergy between microbiota diversity and neuronal processes.

Although more studies on brain-gut-microbiota communication are necessary for establishing effective therapies for CNS disorders, multidisciplinary approaches provide valuable insights and sustain the development of future treatments (Grenham et al., 2011). Furthermore, the specific bacterial species most significantly altered during AD progression remain uncertain, highlighting the need for continued research to effectively utilize bacterial groups as biomarkers in early diagnosis. Investigating the correlation between bacterial metabolites, such as acetate, and taxonomic composition data (Ferreiro et al., 2023) could clarify the role of specific gut taxa in AD.

## 4 *Drosophila* as a gut-brain axis model

*Drosophila* is frequently used in genetic research, and its tractable microbiome makes it a valuable axenic and gnotobiotic model (Brummel et al., 2004; Steven et al., 2021). This allows controlled interactions between the host and known microorganisms, which can be useful in assessing aggressive behaviors (Jia et al., 2021) and locomotion patterns (Schretter et al., 2018). With a relatively simple microbiota (Marra et al., 2021), *D. melanogaster* holds microbial communities of 2 to 30 species, that are represented by two phyla: *Proteobacteria* and *Firmicutes*. The most consistently associated species across different studies are lactic and acetic acid bacteria that reflects the fermentative substrates on which flies feed (Arias-Rojas and Iatsenko, 2022).

The intestine of *Drosophila* exhibits well-conserved molecular aspects with humans (Apidianakis and Rahme, 2011) and distinct pH zones (Sapre et al., 2020), making it a widely used model in gut-related studies (Iatsenko et al., 2018; Dodge et al., 2023; Silva et al., 2020). The gastrointestinal tract is divided into the foregut, midgut and hindgut, with the midgut harboring the gastric acid-producing copper cells (Miguel-Aliaga et al., 2018; Broderick and Lemaitre, 2012) (Figure 1C), which, similarly to the human stomach, may affect pH-sensitive bacteria and influence the microbiota composition (Storelli et al., 2018). The *Drosophila* gut is altered by the ingestion of *Pseudomonas entomophila* (Vodovar et al., 2005) and *Erwinia carotovora* (Buchon et al., 2009), which influence the cytoskeleton composition of gut epithelial cells and promote intestinal stem cell proliferation, respectively. Additionally, the gut epithelium secretes a mucus layer and the chitin-based peritrophic matrix, which act as filters for pathogenic microorganisms (Vodovar et al., 2005; Apidianakis and Rahme, 2011).

Species such as *Acetobacter fabarum* and *Lactobacillus brevis* assist in *Drosophila* nutrition (Sommer and Newell, 2019), while microbiota-derived acetate activates intestinal innate immunity (Jugder et al., 2021). Furthermore, *Lactobacillus plantarum*, a bacterium found in the *Drosophila* intestine, influences larval growth through a nutrient-sensing system (Storelli et al., 2011), and the gut microbiome prevents rapid fluctuations in the circadian cycle of flies (Zhang et al., 2023), reinforcing the communication between the two organs.

In fly models of both AD and Parkinson's disease, the proportion of *Acetobacter* and *Lactobacillus* is lower than in healthy controls (Kong et al., 2018; Liu et al., 2023). Lactic acid is the main metabolite of *Lactobacillus* and stimulates the production of reactive oxygen species (ROS) *via* the intestinal NADPH oxidase Nox (Iatsenko et al., 2018), a process strongly associated with neurodegeneration. Moreover, the *Drosophila*'s metabolism is highly adaptive; when the glycolytic pathway is insufficient, its glial cells can switch to using fatty acids to fuel neuronal metabolism (McMullen et al., 2023), suggesting that these cells contribute to the gut-brain axis as either intermediaries in neurodegeneration or nutrient processing. In summary, both microbial metabolites and the composition of microbial species are strong candidates for contributing to AD progression.

The *Drosophila* gut-brain axis is also reflected in its anatomy, where nerve fibers are regulated by cells in the digestive tract. Serotonergic enterochromaffin cells, a type of cell found in the human gut epithelium, were shown to modulate sensory nerves *via* serotonin receptors and synaptic connections (Bellono et al., 2017). Some subtypes of these enterochromaffin cells are also found in *Drosophila* (Guo et al., 2022), suggesting that flies, like humans, experience environmental, metabolic, and homeostatic signals from the gut directly to their nervous system.

## 5 The CNS glial cells of *Drosophila*

Fruit flies are extensively used as animal models in neurocognitive and physiological experiments (Kitani-Morii et al., 2021). These studies employ various assays, including negative geotaxis (Rahmani et al., 2022; Ferreiro et al., 2018), gastric motility (Rydbom et al., 2021), and memory-related behavior (Gil-Martí et al., 2023). Physiological and behavioral alterations associated with AD can be assessed through multiple methods, such as monitoring sleep (Vaccaro et al., 2020), profiling the transcriptome (Marsh et al., 2016; Zhang et al., 2023; Liu et al., 2023), assessing lifespan (Vaccaro et al., 2020; McMullen et al., 2023), quantifying bacteria (Zhang et al., 2023; Trébuchet et al., 2019), evaluating microglial metabolic alterations (Marsh et al., 2016; Huang et al., 2023), and assessing glial development (Stork et al., 2012).

The *Drosophila* nervous system exhibits a significant level of complexity, sharing cellular, genetic, and functional characteristics with its mammalian counterparts (Salazar et al., 2022). Some authors categorize glial cells into four categories: cortical glia, neuropil glia, peripheral glia, and surface glia (Freeman and Doherty, 2006; Yildirim et al., 2019), but the classification may vary depending on characteristics such as cell body position and form. A comprehensive classification is presented in Table 1, considering the morphological and functional similarities of glial subtypes.

**Table 1:** Types and functions of glial cells in the adult *Drosophila* CNS.

**Glial type**	**Function**	**Position**
**Astrocyte-like**	**Ionic and neurotransmitters homeostasis**	**Outside (cell bodies) and inside (extended processes) the neuropil**
Ensheathing	Phagocytosis of debris	Between neuropil surface and cortex cells
Cortex	Trophic support to neurons	CNS cortex
Subperineural	Chemo-protection and selective transport of nutrients	CNS periphery
Perineural	Chemo-protection, selective transport of nutrients, and barrier physical support	Covering the entire nervous system

The evolution of the nervous system has resulted in a higher proportion of glial cells compared to neurons, with estimates of 15% in flies, 50% in mice, and 90% in humans, indicating an increasing contribution of glia according to complexity (Kremer et al., 2017). Similar to mammalian microglia, the surface and neuropil glia of *Drosophila*—specifically, the ensheathing glia—perform macrophage-like functions (Freeman and Doherty, 2006), suggesting their involvement in the engulfment of Aβ and tau fragments.

The perineural and subperineural glia perform a blood-brain barrier role in *Drosophila*, controlling the passage of bacterial metabolites to the brain. Furthermore, glial cells, such as astrocytes, supply lactate to neurons (Hascup et al., 2022), a function of cellular cooperation that is also conserved in *Drosophila* (Volkenhoff et al., 2015), but is abnormally altered in energy-demanding neurons affected by AD. The variety of transgenic lineages and the ease of using flies as axenic and gnotobiotic models make this organism useful in researching neurological conditions as diverse as AD and autism (Salim et al., 2021).

Similar to the mammalian vagus nerve, *Drosophila* gut-brain communication is mediated by serotonergic neurons that innervate its intestine (Schoofs et al., 2014). This enteric nervous system of the invertebrate model—including both neurons and glial cells—connects to the central nervous system *via* the antennal nerve (Salim et al., 2021; Schoofs et al., 2014), a crucial pathway for transporting bacterial metabolites across the gut-brain axis. The relatively small number of glial cells in flies, compared to mammals, may offer a unique opportunity to better understand glial communication with the neuronal microenvironment.

Intriguingly, when AD disrupts the gut microbiota of mammals, *Lactobacillus* produces such high levels of GABA that the mucin layer is compromised, allowing the movement of solutes and metabolites out of the intestine (Conn et al., 2024). Furthermore, enteric glial cells of mice express GABA signaling receptors (Deng et al., 2023), raising the question of whether a similar host-bacteria communication could occur in *Drosophila*, a model organism with well-characterized genome and largely mapped neuronal connectome.

In mammals, the neuroinflammation related to AD is intricately associated with microglial activation (Johnson et al., 2020), which causes the cell to undergo a morphological transformation from a slender, ramified form to a more rounded shape with fewer extensions (Loh et al., 2024; Colombo et al., 2021). In addition, the gut microbiome of mice has been shown to modulate the expression of AD-related genes, such as *Apoe* and *Trem2* (Huang et al., 2023). In flies, the interaction of neurons with their support cells and the expression of genes involved in Aβ clearance in glial cells (Yang et al., 2017) warrant a deeper investigation into the molecular mechanisms underlying gut-brain axis.

## 6 Discussion

The indirect mechanisms linking intestinal dysbiosis to the progression of Alzheimer's disease remain poorly understood, with most current studies primarily focusing on direct correlations. The complexity of inter-organ communication and the impact of environmental factors such as stress, sleep, and social interactions on neurocognitive impairment are still in discussion. Employing an *in vivo* system for such investigations could better reflect the complexity of signals that CNS cells receive and process, producing a more representative output that facilitates the development of new therapies.

The well-characterized genome and genetic tools of *Drosophila*, along with its simpler microbiota and low maintenance requirements, serve as motivating reasons to use this model for evaluating the influence of extraneural events on the progression of Alzheimer's disease. The behaviors exhibited by the flies and their metabolic pathways are highly mappable, facilitating the analysis of the interplay among comprehensive and robust hypotheses.

Fruit flies are also an excellent model for developing and testing drugs, though they are still timidly utilized in biotechnology research. Considering the development of probiotics, the modifiable microbiome of *Drosophila* could accelerate the creation of new medications and improve safety before clinical trials. When used in pioneering research and in association with complementary models, *Drosophila* can foster new discoveries in the gut-brain axis field, translating evolutionarily conserved associations into theragnostic solutions, from bench to bedside.
